# Cardiac arrest due to an anomalous aortic origin of a coronary artery: are older patients really safe?

**DOI:** 10.5935/0103-507X.20200099

**Published:** 2020

**Authors:** Inês Almeida, Helder Santos, Hugo Miranda, Mariana Santos, Samuel Almeida, Joana Chin

**Affiliations:** 1 Centro Hospitalar Barreiro Montijo EPE - Barreiro, Portugal.

**Keywords:** Hemodynamics, Heart arrest, Coronary artery, Hemodinâmica, Parada cardíaca, Artéria coronária

## Abstract

The authors report a rare case of successful Advanced Life Support in the context of cardiac arrest due to the presence of an anomalous aortic origin of the right coronary artery in a 49-year-old patient. The patient was admitted due to chest pain and dyspnea, with rapid evolution of pulseless ventricular tachycardia and cardiopulmonary arrest. Acute myocardial infarction was considered, and in the absence of a hemodynamic laboratory in the hospital, thrombolysis was performed. Subsequently, coronary angiography revealed no angiographic lesions in the coronary arteries and an anomalous right coronary artery originating from the opposite sinus of Valsalva. Coronary computed tomography angiography confirmed this finding and determined the course between the pulmonary artery and the aorta. The patient underwent cardiac surgery with a bypass graft to the right coronary artery, with no recurrent episodes of arrythmia.

## Abstract (pt)

Os autores relatam um raro caso de uso de Suporte Avançado de Vida no contexto de uma parada cardíaca ocorrida em razão de uma origem aórtica anômala da artéria coronária direita em um paciente de 49 anos de idade. O paciente foi admitido com dor torácica e dispneia, evoluindo rapidamente para taquicardia ventricular sem pulso e parada cardiopulmonar. Considerou-se um infarto agudo do miocárdio e, na ausência de um laboratório de hemodinâmica no hospital, realizou-se trombólise. Subsequentemente, uma angiografia coronária revelou ausência de lesões angiográficas nas artérias coronárias e origem anômala da artéria coronária direita do seio de Valsalva oposto. Uma angiografia coronária por tomografia computadorizada confirmou o achado e determinou um trajeto entre a artéria pulmonar e a aorta. O paciente foi submetido à cirurgia cardíaca com realização de ponte de mamária para a artéria coronária direita, sem qualquer novo episódio de arritmia.

## INTRODUCTION

An anomalous aortic origin of a coronary artery (AAOCA) is rare, with an estimated incidence of 1%, and there is no sex prevalence.^(1,2)^ Although most of cases are asymptomatic and benign, approximately 20% lead to myocardial ischemia, arrhythmia and death.^(1,3,4)^ Chest pain, dyspnea, dizziness, syncope and sudden cardiac arrest, typically during exercise, may be the presentation of AAOCAs. This clinical expression is most common between 10 and 25 years, but symptoms can occur even at rest after the age of 40 - 50 years (possibly because of aortic root dilatation and worsening stenosis).^(5)^ An AAOCA is the second leading cause of sudden cardiac death in apparently healthy young athletes after hypertrophic cardiomyopathy.^(2,6,7)^ Angina is the most frequent indication for coronary angiography (43.9%).^(1)^

An AAOCA can be classified according to the ectopically originating coronary artery (with the circumflex artery being the most common, followed by the single coronary artery) or the ectopic origin site.^(1)^ An ectopic aortic origin is the most common type, of which an anomalous origin from an incorrect sinus of Valsalva is predominant.^(1,8)^ The high-risk anatomies are a coronary artery segment coursing between the pulmonary artery and aorta, which dilate during exercise and thus compress the coronary artery; an acute angle of the initial trajectory of the coronary artery; ostial abnormalities; an intramural course (course within the aortic wall prior to exiting the mediastinum); and vessel spasms and intussusception of the anomalous artery.^(1,2,9-11)^ An anomalous origin of the right coronary artery from the left coronary sinus is frequently associated with exercise-related sudden death.^(1)^

Diagnosis of an AAOCA may be suggested with transthoracic or transesophageal echocardiography by abnormal biphasic flow in the left ventricular outflow tract.^(1)^ Coronary angiography, coronary computed tomography angiography (CCTA) and cardiac magnetic resonance (CMR) allow direct visualization of an AAOCA, establishing the diagnosis.^(1)^ The preferred method, due to its noninvasive nature and acceptable availability, in addition to its precise demonstration of the anatomy and course of the anomalous vessel, is CCTA.^(4,12,13)^

The treatment of asymptomatic patients, especially those with an anomalous right coronary artery from the left sinus of Valsalva with an interarterial or intramural course, is controversial among authors.^(11)^ If associated with symptoms (chest pain or syncope) or documented ischemia during stress tests and/or inducible myocardial perfusion abnormalities on advanced imaging, surgical correction is recommended according to the guidelines for the management of adults with congenital heart disease published in 2008 by the American College of Cardiology/American Heart Association (class indication I, evidence level B).^(2,14,15)^ Sudden death is rare in individuals older than 30 years. In this population, the risk associated with surgery exceeds the potential benefits, and surgery should be avoided in both asymptomatic and symptomatic individuals unless ischemia can be documented.^(2)^^)^ Surgical interventions, most commonly coronary translocation, are safe, with a low incidence of mortality and postoperative complications.^(16,17)^ Coronary artery bypass grafts should be avoided, especially in younger populations, given the potential for competitive flow from native vessels to cause graft failure. There are a few reports of percutaneous coronary intervention of anomalous coronary arteries with good results, even in critically ill newborns for whom there were no other treatment options. However, the insertion of stents into coronary arteries in young patients can be problematic.^(5,14)^

## CASE DESCRIPTION

The authors present the case of a 49-year-old male patient. He was previously asymptomatic and had no relevant personal background or chronic medication. He was a current smoker but denied either alcoholic or toxic consumption. He was admitted to the emergency room due to oppressive chest pain and dyspnea that started half an hour before. Five days prior to hospital admission, the patient reported a similar episode of oppressive chest pain that started during a walk and lasted for approximately 30 minutes after resting.

Upon arrival at the emergency room, the patient was prostrated and cyanotic, with an increased respiratory rate and hypoxia despite a high debit oxygen mask. The patient evolved rapidly to cardiopulmonary arrest, so advanced life support maneuvers were initiated, and the patient was orotracheally intubated and invasively ventilated. Blood gas analysis showed severe metabolic acidosis with hyperlacticaemia (pH = 6.87; partial pressure of carbon dioxide - PaCO_2_ - 105mmHg, partial pressure of oxygen - PaO_2_ - 120mmHg, bicarbonate - HCO_3_ - 13.9, lactate 13.5mmol/L). An electrocardiogram revealed R-R regular wide complex tachycardia (170/minute) with right axis deviation, suggestive of ventricular monomorphic tachycardia. Since there was no response to advanced life support maneuvers with persistent pulseless ventricular tachycardia, and considering the patient’s complaints of chest pain, acute myocardial infarction was considered, and thrombolysis with alteplase was performed in the absence of a hemodynamic laboratory in the hospital. After recovery to spontaneous circulation, an electrocardiogram showed sinus rhythm, 90/minute, biphasic T between V3 and V5.

The patient was transferred to the intensive care unit. A transthoracic echocardiogram showed mild hypokinesis of the apical segment of the inferolateral wall. Blood analysis revealed mild anemia (hemoglobin 11.5g/dL), acute kidney injury (creatinine 2.26mg/dL and urea 76mg/dL) and mild hypokalemia (3.2mmol/L). Myocardial necrosis markers were mildly elevated: *hs*-troponin 84pg/mL (cutoff 34.2pg/mL), creatinine kinase 510UI/L (cutoff 200UI/L), creatinine kinase - MB 6.8ng/mL (cutoff 7.2ng/mL) and myoglobin 2914ng/mL (cutoff 116ng/mL). The D-dimer level was also elevated (1,678ng/mL, cut off 500ng/mL). *Hs*-troponin increased to 358.50pg/mL at 3 hours, and its maximum value was 832pg/mL, 24 hours after admission.

Thrombolysis was complicated by bleeding at the central venous catheter puncture site and abundant hematemesis. Upper digestive endoscopy was performed, showing bleeding of the gums, digested blood and clots in the stomach. Due to active bleeding, the case was discussed in hemodynamic laboratory, and coronary angiography was delayed until clinical stabilization and bleeding cessation occurred.

**Figure 1:**
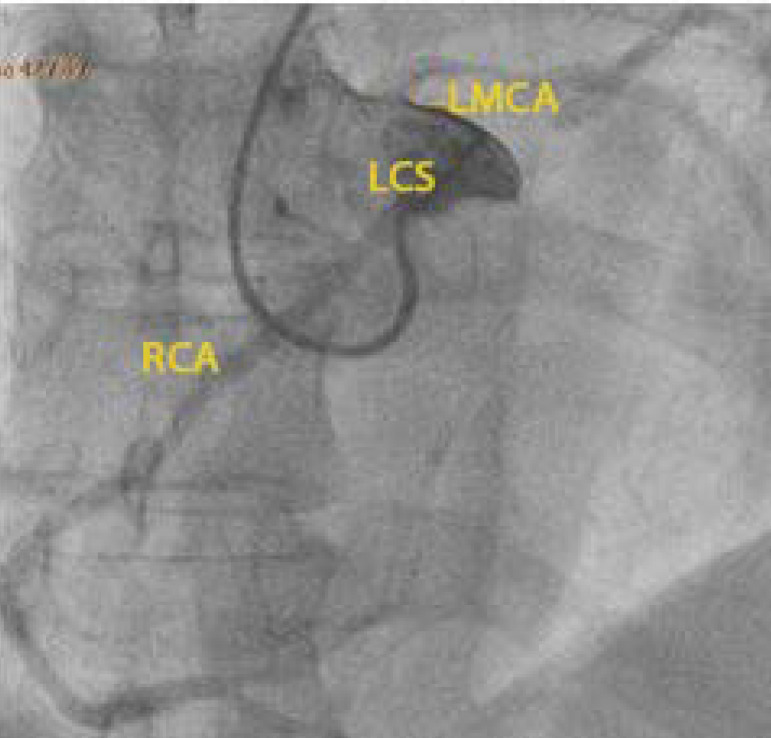
Coronary angiography showed a right coronary artery with an abnormal origin at the left coronary sinus.

**Figure 2:**
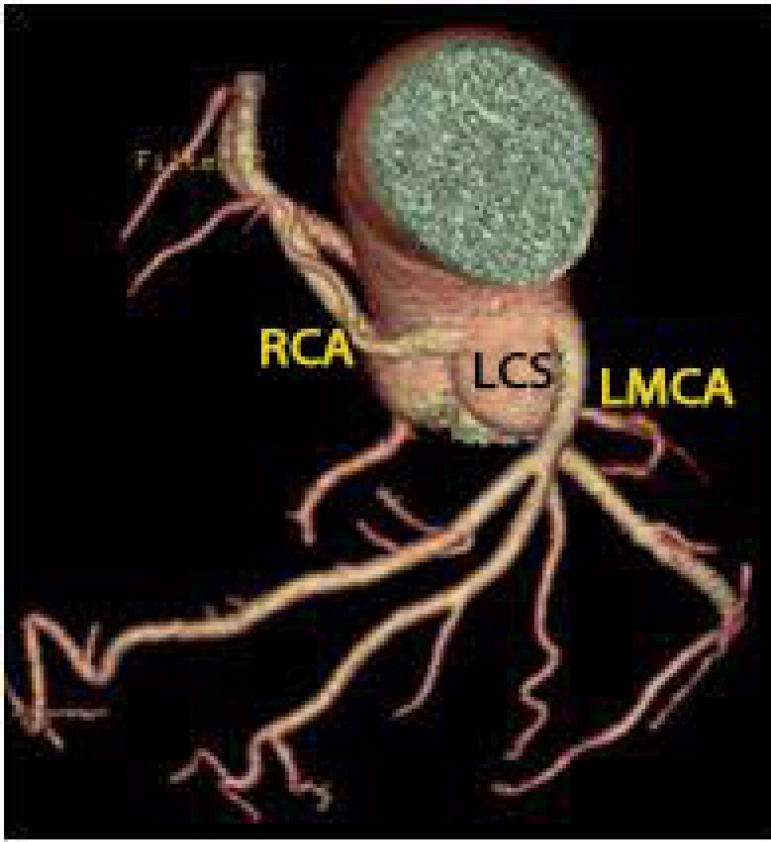
Coronary computed tomography angiography confirmed the abnormal origin of the right coronary artery from the left coronary sinus.

**Figure 3:**
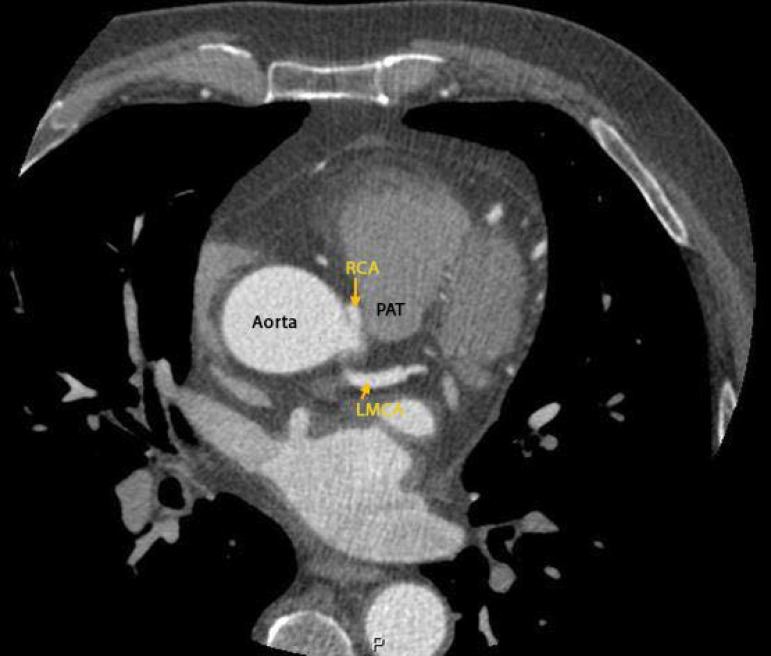
Coronary computed tomography angiography confirmed the abnormal origin of the right coronary artery from the left coronary sinus coursing between the pulmonary artery trunk anteriorly and the aorta posteriorly.

Three days after admission, the patient progressively improved, allowing for orotracheal extubation. Brain computed tomography was unremarkable. A ventilation-perfusion scan was performed, excluding pulmonary embolism. Coronary angiography showed coronary arteries with no relevant angiographic lesions and a right coronary artery with an abnormal origin at the left coronary sinus (Figure 1), followed by CCTA that confirmed the abnormal origin of the right coronary artery from the left coronary sinus coursing between the pulmonary artery trunk anteriorly and the aorta posteriorly - an interarterial course with an extension of 18mm; there was no evidence of an intramural course or atherosclerotic coronary disease, with a calcium score 0 (Figures 2 and 3). Cardiac magnetic resonance was unremarkable - no late enhancement was detected.

LCS - left coronary sinus; LMCA - left main coronary artery; RCA - right coronary artery.

LCS - left coronary sinus; LMCA - left main coronary artery; RCA - right coronary artery.

RCA - right coronary artery; LMCA - left main coronary artery; PAT - pulmonary artery trunk.

## DISCUSSION

The most likely causes of cardiopulmonary arrest in the present case are acute coronary syndrome and massive pulmonary embolism. With only mild elevation of *hs*-troponin in a clinical setting of cardiorespiratory arrest, the absence of lesions on coronary angiography, even after thrombolysis, and the absence of late enhancement on cardiac magnetic resonance make the hypothesis of myocardial infarction unlikely. Although fibrinolysis is only indicated in patients presenting early and when anticipated ST elevation myocardial infarction diagnosis and the mediated reperfusion time is more than 120 minutes - it was performed in this case due to the inexistence of a hemodynamic laboratory in our hospital and the clinical instability that did not allow transportation to the primary percutaneous coronary intervention center. Prolonged resuscitation may increase bleeding risks and may be a relative contraindication to fibrinolysis; however, it is important to weigh the potentially life-saving effect of fibrinolysis against the potentially life-threatening side effects. This must also be weighed against the risks and benefits of delayed primary percutaneous coronary intervention. The hypothesis of a massive pulmonary embolism was also excluded in the absence of dilation and dysfunction of the right ventricle on the transthoracic echocardiogram and the absence of perfusion defects on the ventilation-perfusion scan (although it was performed after thrombolysis). Other causes, such as electrolyte disturbances, drug consumption, myocarditis and hypertrophic cardiomyopathy, were also excluded. In this case, CCTA confirmed the presence of an anomalous right coronary artery with an interarterial course. Based on the clinical history and the results of complementary diagnostic exams, the diagnosis of an abnormal coronary origin responsible for cardiorespiratory arrest was considered, and the patient was referred for cardiac surgery. He successfully underwent myocardial revascularization surgery with a bypass from the left internal mammary artery to the right coronary artery. After cardiac surgery, the patient stayed clinically stable, with no recurrent episodes of arrythmia.

Despite an extensive literature review, there appear to be just two documented cases in the European Heart Journal and British Medical Journal of survivors of cardiac arrest due to an anomalous right coronary artery documented by CCTA; these patients proceeded to undergo successful surgical correction.^(18,19)^ The vast majority of other documented cases are from necropsy case series.

## CONCLUSION

An anomalous aortic origin of a coronary artery is rare, and most cases are asymptomatic and benign. Its presentation, most commonly occurring between 10 and 25 years, may include chest pain, syncope and sudden cardiac arrest, typically during exercise. The treatment of asymptomatic patients, especially those with an anomalous right coronary artery, is controversial if symptomatic surgical correction is recommended. Our case report highlights the fact that, although exceedingly rare in older adults, sudden cardiac arrest may occur in the setting of an anomalous aortic origin of a coronary artery.
